# Inhibiting glutamine utilization creates a synthetic lethality for suppression of ATP citrate lyase in KRas-driven cancer cells

**DOI:** 10.1371/journal.pone.0276579

**Published:** 2022-10-21

**Authors:** Ahmet Hatipoglu, Deepak Menon, Talia Levy, Maria A. Frias, David A. Foster

**Affiliations:** 1 Department of Biological Sciences, Hunter College of the City University of New York, New York, New York, United States of America; 2 Biochemistry Program, Graduate Center of the City University of New York, New York, New York, United States of America; 3 Department of Biology and Health Promotion, St Francis College, Brooklyn, New York, New York, United States of America; 4 Biology Program, Graduate Center of the City University of New York, New York, New York, United States of America; 5 Department of Pharmacology, Weill Cornell Medicine, New York, New York, United States of America; Emory University, UNITED STATES

## Abstract

Metabolic reprogramming is now considered a hallmark of cancer cells. KRas-driven cancer cells use glutaminolysis to generate the tricarboxylic acid cycle intermediate α-ketoglutarate via a transamination reaction between glutamate and oxaloacetate. We reported previously that exogenously supplied unsaturated fatty acids could be used to synthesize phosphatidic acid–a lipid second messenger that activates both mammalian target of rapamycin (mTOR) complex 1 (mTORC1) and mTOR complex 2 (mTORC2). A key target of mTORC2 is Akt–a kinase that promotes survival and regulates cell metabolism. We report here that mono-unsaturated oleic acid stimulates the phosphorylation of ATP citrate lyase (ACLY) at the Akt phosphorylation site at S455 in an mTORC2 dependent manner. Inhibition of ACLY in KRas-driven cancer cells in the absence of serum resulted in loss of cell viability. We examined the impact of glutamine (Gln) deprivation in combination with inhibition of ACLY on the viability of KRas-driven cancer cells. While Gln deprivation was somewhat toxic to KRas-driven cancer cells by itself, addition of the ACLY inhibitor SB-204990 increased the loss of cell viability. However, the transaminase inhibitor aminooxyacetate was minimally toxic and the combination of SB-204990 and aminooxtacetate led to significant loss of cell viability and strong cleavage of poly-ADP ribose polymerase–indicating apoptotic cell death. This effect was not observed in MCF7 breast cancer cells that do not have a KRas mutation or in BJ-hTERT human fibroblasts which have no oncogenic mutation. These data reveal a synthetic lethality between inhibition of glutamate oxaloacetate transaminase and ACLY inhibition that is specific for KRas-driven cancer cells and the apparent metabolic reprogramming induced by activating mutations to KRas.

## Introduction

Mutant KRas-driven cancers represent as much as one third of all human cancers [1]. Inhibiting KRas directly in these cancers has proved to be difficult–suggesting that it might be wiser to attack KRas signals indirectly. A fertile area for targeting KRas indirectly is the metabolic reprogramming observed in KRas-driven cancer cells [1]. KRas-transformed cells have an addiction to glutamine (Gln) and use it as an important source for both carbon and nitrogen [2–5]. We reported previously that blocking anaplerotic entry of Gln into the tricarboxylic acid (TCA) cycle sensitizes KRas-driven cancer cells to rapamycin and other cytotoxic drugs that target cells in S-phase of the cell cycle [6–8]. Anaplerotic entry of Gln into the TCA cycle was via glutaminolysis whereby Gln is deamidated to glutamate and then deaminated via glutamate oxaloacetate transaminase (GOT), where glutamate is converted to α-ketoglutarate (α-KG) and oxaloacetate (OAA) is converted to aspartate [2, 6]. The logic behind the generation of α-KG from Gln rather than from citrate generated from the condensation of OAA with acetyl-coenzyme A (acetyl-CoA) from glycolysis is that citrate generated from glucose can now exit the mitochondria and be converted back into acetyl-CoA and OAA by ATP citrate lyase (ACLY) [9]. In this way, the reduced carbon of acetyl-CoA can be used in the cytosol for anabolic reactions such as fatty acid synthesis rather than used up by a dehydrogenase reaction to generate CO_2_ and NADH. Thus, ACLY is in a position to regulate metabolic flux between catabolic metabolism in the mitochondria and anabolic metabolism in the cytosol [9, 10]. Acetyl-CoA metabolism has been implicated in multi-step pancreatic cancer, which is driven by KRas [11].

We reported previously that exogenously supplied oleic acid (OA), a mono-unsaturated fatty acid, could lead to the generation of phosphatidic acid (PA), which can activate mTOR complexes 1 and 2 [12]. mTORC2 localizes to mitochondria-associated endoplasmic reticulum membranes (MAMs) [13, 14]. The mTORC2-Akt signaling node at the MAMs regulates mitochondrial physiology [14]. Akt phosphorylates ACLY at S455 [15]–suggesting that the OA-induced phosphorylation of Akt at the mTORC2 site at S473 could also lead to the phosphorylation of ACLY and regulation of metabolic flux.

In this report, we demonstrate that the OA-induced phosphorylation of ACLY at the Akt phosphorylation site at S455 occurs in an mTORC2-dependent manner. Mutant KRas-driven cancer cells are dependent on ACLY for viability in the absence of serum or Gln, while non-KRas-driven cancer cells and normal cells studied did not demonstrate this dependency. Interfering with the transamination reaction responsible for the generation of α-KG from glutamate created a synthetic lethality with ACLY inhibition. Inhibition of transamination or ACLY is well tolerated in mice [10, 16, 17]. This suggests that inhibition of GOT along with ACLY inhibition could be a viable therapeutic option for treating KRas-driven cancers where there is a dependence on GOT to generate α-KG. α-KG can then be used to generate OAA that condenses with acetyl-CoA from glycolysis to generate more citrate [18].

## Materials and methods

### Reagents and antibodies

The ACLY inhibitor SB-204990 (Sigma SML2829), OA (Sigma O3008), fatty acid-free bovine serum albumin (BSA) (Sigma A9205), dimethyl α-ketoglutarate (DMKG) (Sigma 349631) were purchased from Sigma. Antibodies against Rapamycin-insensitive companion of mammalian target of rapamycin (Rictor) (9476), P-ACLY [S455] (4331), P-Akt [473] (9271), cleaved poly-ADP-ribose polymerase (PARP) (9541), and Actin (3700) were obtained from Cell Signaling Technology. Anti-mouse (SA00001-1) and anti-rabbit (SA00001-2) HRP-conjugated secondary antibodies were obtained from Proteintech. All other reagents, unless noted otherwise, were purchased from Fisher Scientific.

### Cells and cell culture conditions

HCT116, MDA-MB-231, MCF7, and BJ-hTert cells used in this study were obtained from the American Type Tissue Culture Collection. HCT116 cells were maintained in Dulbecco’s Modified Eagle Medium (DMEM) (Sigma D5796) supplemented with 10% Fetal Bovine Serum (FBS) (Sigma 12306C). All other cells were maintained in DMEM (Sigma D6429) supplemented with 10% FBS (Sigma 12306C). Media used for glutamine free conditions were DMEM (Sigma D5671) for HCT116 cells and DMEM (Sigma D5546) for all other cell lines. All culture media was supplemented with 1x antibiotic/antimycotic solution (Sigma A5955). No further authentication was performed.

### Cell viability

(2,3-bis-(2-methoxy-4-nitro-5-sulfophenyl)-2H-tetrazolium-5-carboxanilide) (XTT) reduction assay was used to measure cells viability. In brief, 5×10^4^ cells/500 μl/well were seeded into 24-well plates in triplicates. After 24 hours, cells were given treatment medium containing 40 μM ACLY inhibitor (ACLYi)—SB-204990, or vehicle only and incubated at 37°C with 5% CO_2_. After a 24 hr incubation period, cells were treated with 125 μl XTT (Invitrogen^™^ Molecular Probes^™^ XTT (x6493) for 2 hr. Then, plates were read at 450 nm wavelength by a spectrophotometer (Molecular devices, SpectraMax i3). After subtracting blank well absorbance, the absorbance of vehicle treated cells was set to 100%, and the relative absorbance of ACLYi treated cells was reported as % viable cells.

### Transient transfections

Transient siRNA transfections were carried out as described previously [19]. Cells were plated in 6-well plates in complete medium containing 10% FBS. The following day, transfections with siRNA (100 nM) in Lipofectamine RNAiMAX (Thermo Fisher Scientific 137780) were performed in Opti-MEM reduced serum media (Thermo Fisher Scientific 31985070). After 6 hours, cells were given 10% FBS, and the following day, the cells were shifted to complete media with 10% FBS. Cells were then allowed to incubate for indicated times for each experiment, as described in the figure legends. siRNAs were used in the study: Rictor siRNA (Santa Cruz Biotechnology sc-61478), lysophosphatidic acid acyltransferase-β (LPAAT-β) siRNA (Dharmacon M003811) and non-targeting siRNA (Dharmacon D-001206-13).

### Immunoblot analysis

Proteins from the treated cell samples were extracted after lysis with M-PER buffer (ThermoScientific 78501). 20 μg of protein samples were subjected to SDS-PAGE separating gels. Electrophoresed proteins were transferred onto nitrocellulose membrane. Proteins were transferred to nitrocellulose membranes, which were then blocked using 5% nonfat dry milk in phosphate buffered saline containing 0.1% Tween 20 and incubated overnight with primary antibodies as indicated in the text. Membranes were then washed and incubated with secondary antibodies (anti-mouse or anti-rabbit depending on the origin of the primary antibodies) for 1 hr at room temperature and protein levels were visualized using ECL system (Kindle Biosciences 1002).

### Statistical analysis

All statistical details (i.e.: sample size, P-value summaries) of experiments can be found in the figures and their corresponding figure legends. Statistical significance was analyzed using Graph Pad Prism 8 software. For comparisons between two sets of samples, an unpaired t-test was performed. For multiple comparisons, an unpaired, one-way ANOVA analysis was performed. All data is plotted as the mean with the standard deviation. P-value asterisks are defined as follows: *P≤0.05; **P≤0.01; ***P≤0.001; ****P≤0.0001. Not significant (ns) means P>0.05.

## Results

### OA induces ACLY phosphorylation in an mTORC2 dependent manner

**Fig 1:**
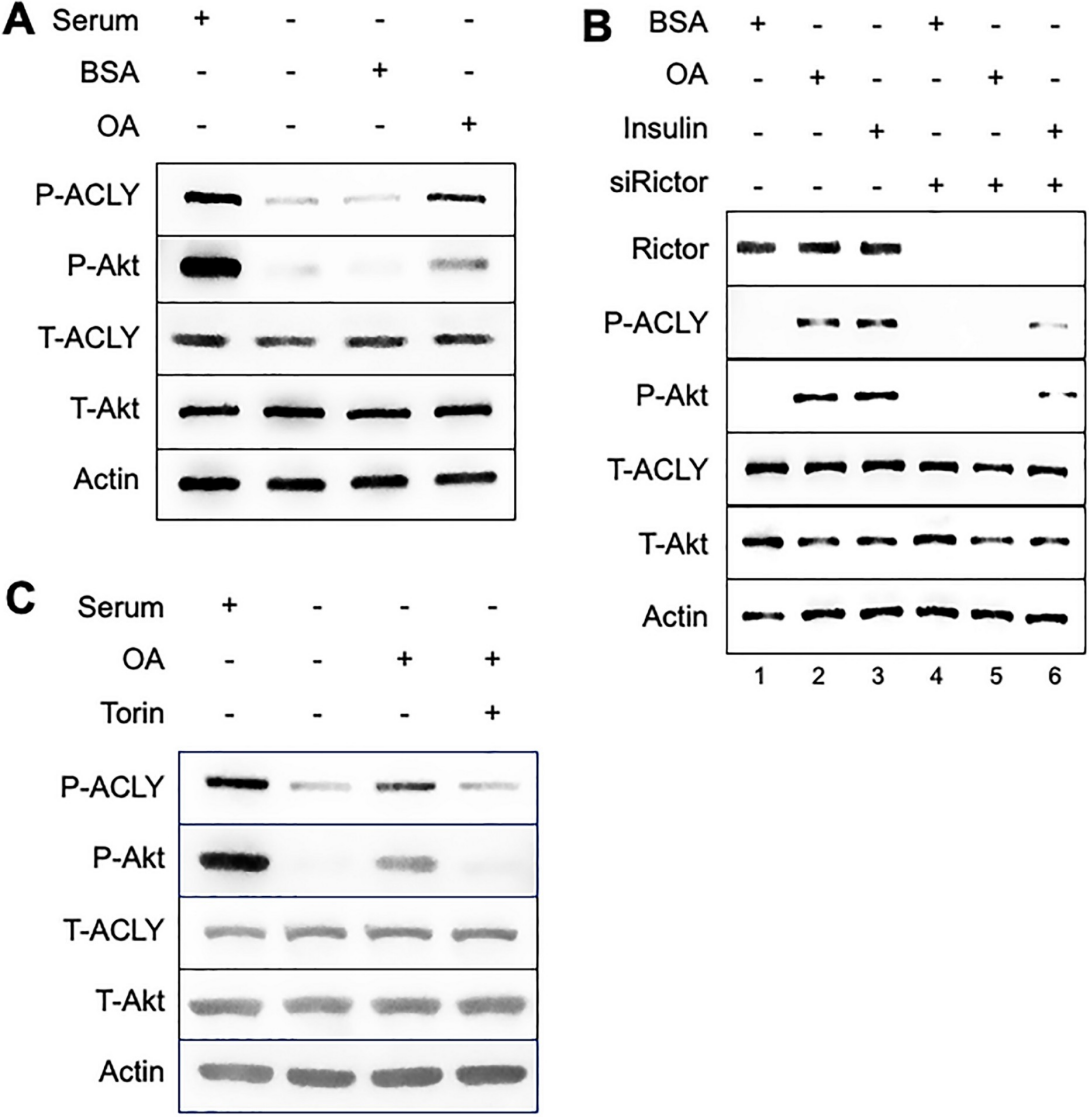
Oleic acid induces ACLY phosphorylation in an mTORC2 dependent manner. (**A**) HCT116 cells were plated at 60% confluency in complete medium (10% serum) and shifted to serum-free medium for 17 hr. OA (10 μM):BSA in a 2:1 ratio was added for 60 min. Fatty acid-free BSA was used as a negative control since OA was carried in complex with BSA. Lysates were collected and analyzed for phosphorylated Akt (S473) and ACLY (S455) via Western blot. (**B**) HCT116 cells were plated at 50% confluency in complete medium. Knockdown of Rictor was carried out via Rictor siRNA (100 nM) (+) or non-targeting (-) siRNA for 72 hr. At the 48 hr point of knock down, the media was changed to serum free media for overnight. OA (10 μM):BSA in a 2:1 ratio was added in presence or absence of Rictor. Insulin (100 nM) was also added to the HCT116 cells in the presence and absence of Rictor. Lysates were collected 60 min later and probed via Western blot for phosphorylated Akt and ACLY as in (A). (**C**) HCT116 cells were plated and treated as in A. Torin1 (1 μM) was used as an mTORC2 inhibitor. Lysates were collected and analyzed by Western blot. BSA, bovine serum albumin. The Western blots shown are representative of experiments repeated at least three times.

We demonstrated previously that OA induces the production of PA in KRas-driven cancer cells [12], which have been reported to scavenge fatty acids [20, 21]. The PA generated in response to OA led to the activation of mTORC2, which in turn phosphorylated Akt at the mTORC2 site at Ser 473 [12]. mTORC2 has been reported to phosphorylate Akt at mitochondrial-associated endoplasmic reticulum membranes (MAMs) [13, 14]. The MAMs are critical for regulating the metabolic reprogramming that occurs in most, if not all, cancer cells [22–24]. Another key enzyme for regulating metabolic reprogramming is ACLY, which diverts mitochondrial citrate to the cytosol where it is converted to acetyl-CoA and OAA, which are used for fatty acid and nucleotide synthesis, respectively [9]. ACLY is phosphorylated by Akt at S445 [15]. We therefore examined whether OA can stimulate the phosphorylation of ACLY at the Akt phosphorylation site at S455. As shown in Fig 1A, OA induced the phosphorylation of ACLY in the KRas-driven colon cancer cell line HCT116 at S455. These data suggest that there is a kinase cascade whereby mTORC2 is activated by the PA generated in response to exogenously supplied OA. mTORC2 then phosphorylates Akt S473, which in turn, phosphorylates ACLY. To establish that the OA-induced phosphorylation of ACLY was dependent on mTORC2, HCT116 cells were treated with siRNA to knock down the mTORC2 subunit Rictor. As shown in Fig 1B, in cells with suppressed Rictor expression, OA was not able to induce the phosphorylation of either Akt at the mTORC2 site at S473 or ACLY at the Akt site at S455 (compare lanes 1 and 2 with lanes 4) and 5). Since mTORC2 is activated by insulin, we also examined the effect of Rictor knockdown on the induction of Akt and ACLY phosphorylation by insulin. As shown in Fig 1B, Rictor knockdown significantly suppressed insulin-induced Akt and ACLY phosphorylation (compare lanes 3 and 6). These data suggest that mTORC2 is required for OA-induced phosphorylation of ACLY. To further establish that mTORC2 is required for the phosphorylation of Akt and ACLY we examined the effect of the catalytic mTOR inhibitor Torin1 on the OA induction of Akt and ACLY phosphorylation. As shown in Fig 1C, Torin1 inhibited the phosphorylation of both Akt and ACLY at the mTORC2 and Akt phosphorylation sites respectively. Collectively, the data provided in Fig 1 are consistent with a model whereby OA stimulates mTORC2 activity via *de novo* PA synthesis [12], followed by the mTORC2-mediated phosphorylation of Akt, which then phosphorylates ACLY. While the data provided here clearly implicate mTORC2 in the OA-induced phosphorylation Akt and ACLY, our previous work showed that OA could also induce phosphorylation of the mTORC1 substrate S6 kinase [12]. Thus, it is still possible that mTORC1 is involved, but given the subcellular localization of mTORC2 and Akt at the MAMs [13, 14] and ACLY being a substrate of Akt, it is likely that mTORC2 is the more significant participant.

### Phosphorylation of ACLY in response to OA is dependent on *de novo* PA production mediated by LPAAT-β

**Fig 2:**
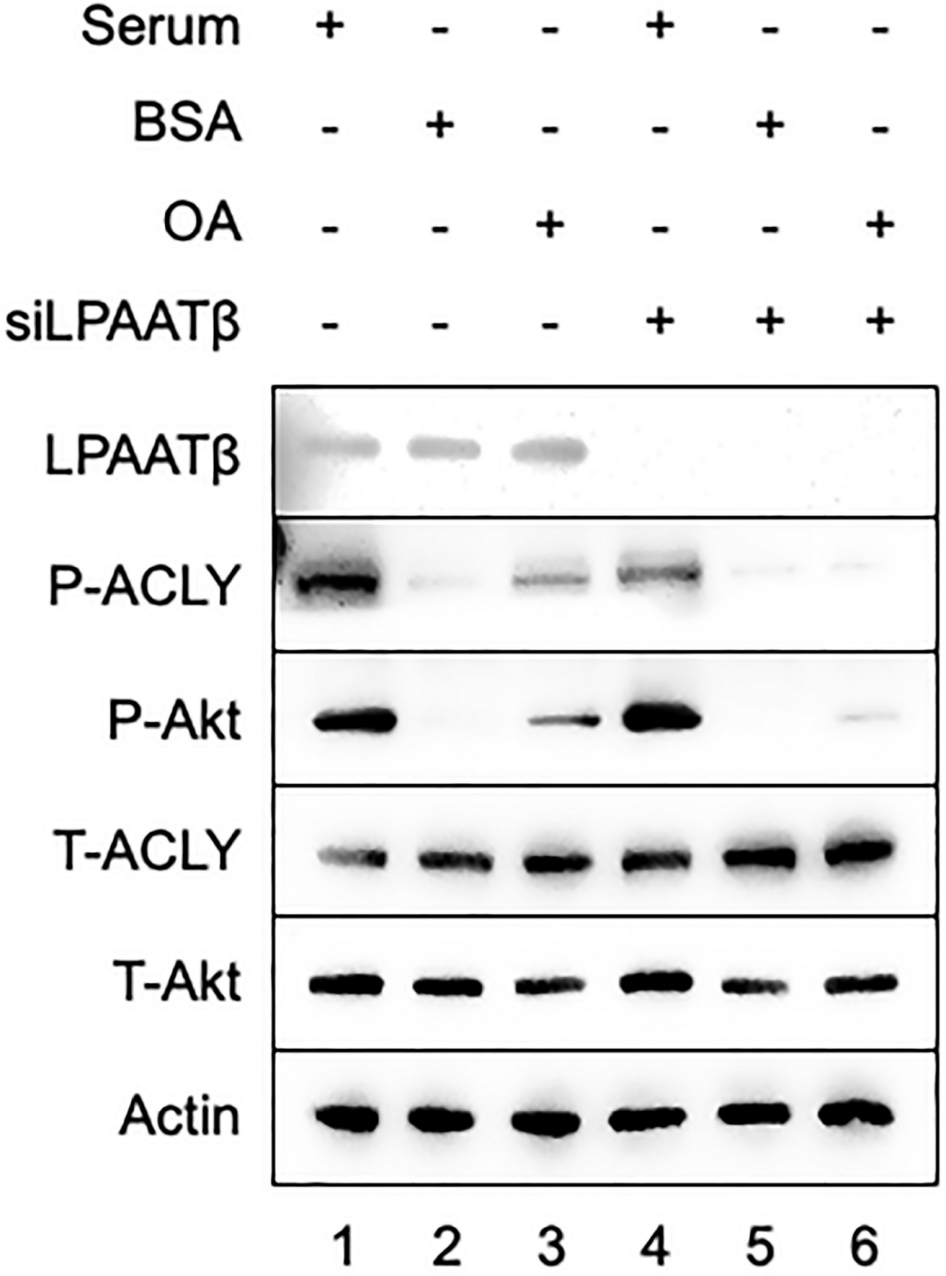
mTORC2 mediated activation of ACLY through production of *de novo* PA in presence of oleic acid. HCT116 cells were plated at 50% confluency. After 24 hr, cells were transfected with LPAAT-β siRNA (100 nM) (+) or non-targeting (-) siRNA for 72 hr. At 48 h point, the media was changed to serum free media for overnight. OA (10 μM):BSA in a 2:1 ratio was added in presence or absence of LPAAT-β. Lysates were collected and analyzed for protein expression. The Western blots shown are representative of experiments repeated at least three times.

To further establish the activation of mTORC2 in response to OA, we examined the dependence of OA-induced phosphorylation of Akt and ACLY on LPAAT-β, the enzyme that catalyzes the acylation of lyso-PA to PA. HCT116 cells were treated with LPAAT-β siRNA to suppress LPAAT-β protein expression. In the presence of LPAAT-β expression, OA induced the phosphorylation of both Akt and ACLY as in Fig 1 (Fig 2). However, in cells with reduced expression of LPAAT-β, OA no longer induced the phosphorylation of Akt and ACLY (compare lanes 2 and 3, with lanes 4 and 5). These results were consistent with our previous report that knockdown of LPAAT-β prevented the OA induction of Akt phosphorylation and PA production [12] and extend the signal stimulated by OA to ACLY phosphorylation at the Akt site at S455 on ACLY.

### KRas-driven cancer cells are sensitive to inhibition of ACLY

**Fig 3:**
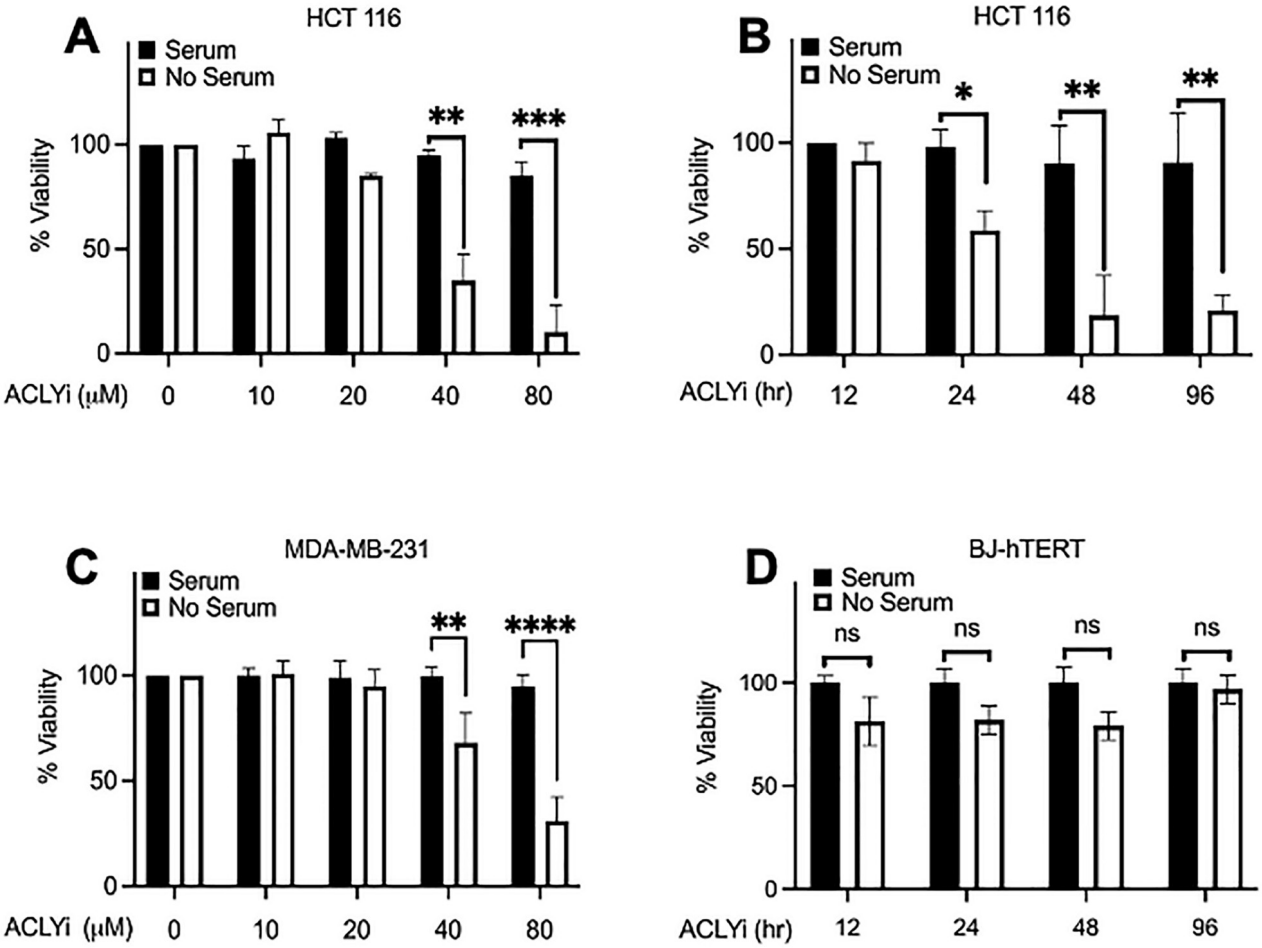
Cytotoxic effect of ACLY inhibition in the absence of serum in KRas-driven cancer cells. (**A**) HCT116 cells were plated at 60% confluence in 96-well plate in complete medium (CM) containing 10% FBS. After 24 hr, cells were shifted to medium with or without serum. Next, cells were transferred into media containing concentrations of 10 μM, 20 μM, 40 μM, and 80 μM ACLYi (SB-204990) for 24 hr. XTT assay was performed for cell viability. (**B**) HCT116 cells were plated as in *A*. After 24 hr, cells were shifted to medium with or without serum. Next, cells were transferred into media containing SB-204990 (40 μM) for 12 hr, 24 hr, 48 hr, and 96 hr time points. XTT assay was performed for cell viability. (**C**) MDA-MB-231 cells were plated and treated as in *A*. XTT assay was performed for cell viability. (**D**) BJ-hTERT cells were plated and treated as in *B*. XTT assay was performed for cell viability. All experiments were repeated two or more times. Error bars represent ± SEM. Unpaired Student’s t-test was performed for statistical analysis.

We reported previously that several human cancer cell lines could be killed by rapamycin in the absence of serum [25, 26]. Since the ability to stimulate ACLY was dependent on mTOR, we examined the impact of an ACLY inhibitor (SB-204990) [10, 27] on cell viability in the presence and absence of serum. HCT116 cells were treated with increasing concentrations of SB-204990 in the presence and absence of serum and cell viability was examined using the XTT assay. As shown in Fig 3A, SB-204990 at 40μM induced loss of cell viability at 24 hr in HCT116 cells deprived of serum. The presence of serum prevented loss of cell viability in the presence of SB-204990. In Fig 3B, a time course experiment indicated that SB-204990 at 40 μM was more effective after 48 hr treatment.

It has been reported that KRas-driven cancer cells scavenge lipids and amino acids [20, 21, 28]. Scavenged fatty acids need to be charged with co-enzyme A (CoA) in order to acylate lyso-PA to generate PA. One of the enzymes that charge fatty acids with CoA is acyl-CoA synthetase long chain 5 (ACSL5). We reported previously that KRas-driven cancer cells express elevated levels of ACSL5 [12]–indicating that KRas triggers the expression of genes involved in *de novo* synthesis of PA and other phospholipids. Two of the KRas driven cancer cells that had elevated ACSL5 were HCT116 and MDA-MB-231 breast cancer cells [12]. We therefore examined whether SB-204990 also induced the loss of cell viability in the MDA-MB-231 cells. As shown in Fig 3C, SB-204990 induced loss of cell viability in the MDA-MB-231 cells similar to that observed with the HCT116 cells (Fig 3A and 3C). We also examined the effect of SB-204990 on the viability of BJ-hTERT human fibroblasts that did not have elevated ACSL5 expression or KRas mutation [12]. As shown in Fig 3D, SB-204990 had no effect on the viability of the BJ-hTERT in the presence or absence of serum. The data in Fig 3 show that inhibition of ACLY in the absence of serum leads to loss of viability in KRas driven cancer cells, but not in the immortal human fibroblast cell line BJ-hTERT.

### Glutamine deprivation sensitizes KRas-driven cancer cells to cytotoxic effects of ACLY inhibition

**Fig 4:**
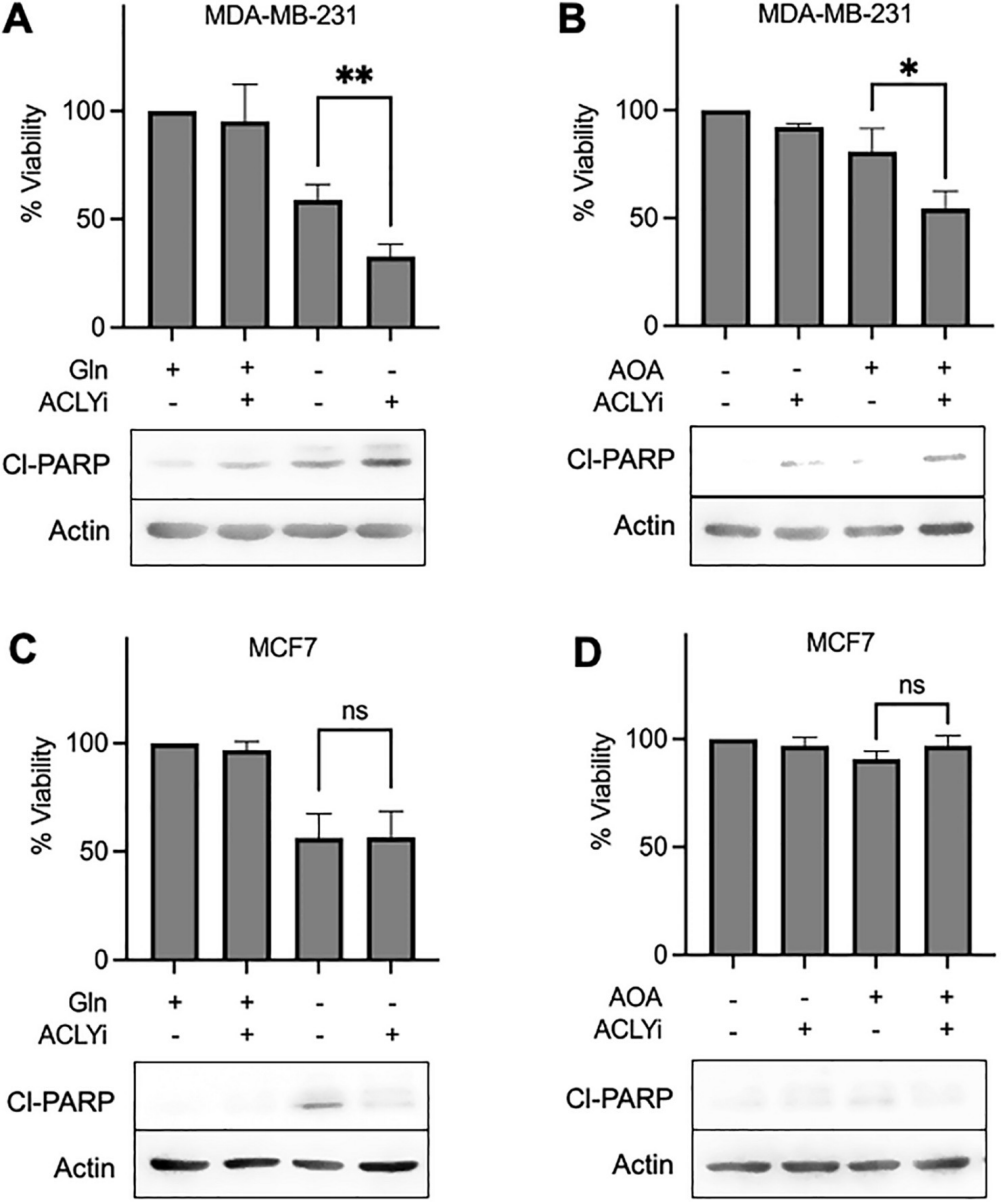
Glutamine deprivation sensitizes KRas-driven cancer cells to ACLY inhibition. (**A**) MDA-MB-231 cells were plated at 60% confluence in 96-well plates in complete medium (CM) containing 10% FBS. After 24 hr, cells were shifted to medium containing or lacking glutamine for 48 hr. ACLY inhibitor (ACLYi) (40 μM) was added for the last 24 hr. Plates were read in a plate reader and analyzed for cytotoxicity via XTT assay. Through concomitant experiments, lysates were collected and analyzed for protein expression via Western blot. (**B**) MDA-MB-231 cells were plated as in *A*. After 24 h, cells were shifted to medium containing or lacking AOA (0.5 mM) for 48 hr. ACLYi (40 μM) was added for the last 24 hr. Plates were read in a plate reader and analyzed for cytotoxicity via XTT assay. Lysates were also collected and analyzed for protein expression via Western blot. (**C**) MCF7 cells were plated and treated as in *A*. Plates were read in a plate reader and analyzed for cytotoxicity via XTT assay. In concurrent experiments, lysates were collected and analyzed for protein expression via Western blot. (**D**) MCF7 cells were plated and treated as in *B*. Plates were read and analyzed for cytotoxicity via XTT assay. Lysates were collected and analyzed for protein expression via Western blot. All experiments were repeated two or more times. Error bars represent ± SEM. Unpaired Student’s t-test was performed for statistical analysis. The Western blots shown are representative of experiments repeated at least two times.

We reported previously that rapamycin killed KRas-driven cancer cells in the absence of Gln [6, 7]. There is a late G1 cell cycle Gln checkpoint [29, 30] that is passed by KRas-driven cancer cells leading to S-phase arrest instead of G1 arrest [6, 7]. Cells arrested in S-phase are sensitive to rapamycin and other drugs that target dividing cells. We therefore investigated whether Gln deprivation impacted the effect of the ACLY inhibitor SB-204990 on KRas-driven cancer cells in the presence of serum. We first investigated the effect of the ACLY inhibitor on cell viability in the presence and absence of Gln in MDA-MD-231 cells in culture medium containing serum. As expected, in the presence of Gln, the ACLY inhibitor had no effect on cell viability (Fig 4A). In the absence of Gln, there was a substantial loss of cell viability. However, SB-204990 treatment led to a 50% further reduction in cell viability (Fig 4A)–indicating that deprivation of Gln increased the sensitivity the cells to SB-204990.

Gln deprivation is not a viable therapeutic option, however it is possible to suppress Gln utilization. A major mechanism for utilizing Gln is via glutaminolysis where Gln is deamidated to glutamate, which is then converted to α-ketoglutarate (α-KG) by dehydrogenation reaction or by transamination with the concomitant conversion of OAA to aspartate [6, 16]. Of significance, KRas-driven cancer cells preferentially use the transamination reaction to generate α-KG [2]. The transamination reaction is catalyzed by GOT, which is inhibited by aminooxyacetate (AOA). AOA has been used in animals without serious side effects [16, 17]. We examined the effect of SB-204990 on MDA-MB-231 cells that were also treated with AOA. As shown in Fig 4B, AOA had substantially less of an effect on viability than did Gln deprivation, and importantly, SB-204990 reduced viability only in cells treated with AOA. We also examined the effect of SB-204990 on the non-KRas-driven breast cancer cell line MCF7 where SB-204990 had no effect on cell viability with either Gln deprivation (Fig 4C) or treatment with AOA (Fig 4D). As with the MDA-MB-231 cells, deprivation of Gln in the MCF7 cells reduced cell viability by itself. But unlike the MDA-MB-231 cells, SB-204990 did not further reduce cell viability in the MCF7 cells (Fig 4C). Surprisingly, SB-204990 actually suppressed PARP cleavage caused by Gln deprivation. While we do not understand why SB-204990 suppresses the PARP cleavage in the MCF7 cells, this observation is consistent with the different metabolism in the KRas-driven cancer cells and the data shown in Fig 4A and 4C. The data in Fig 4 reveal a synthetic lethality for KRas-driven cancer cells between ACLY inhibition and inhibition of Gln utilization via transamination to α-KG.

### α-KG rescues cytotoxic effect of ACLY inhibition upon glutamine deprivation in KRas-driven cancer cells

**Fig 5:**
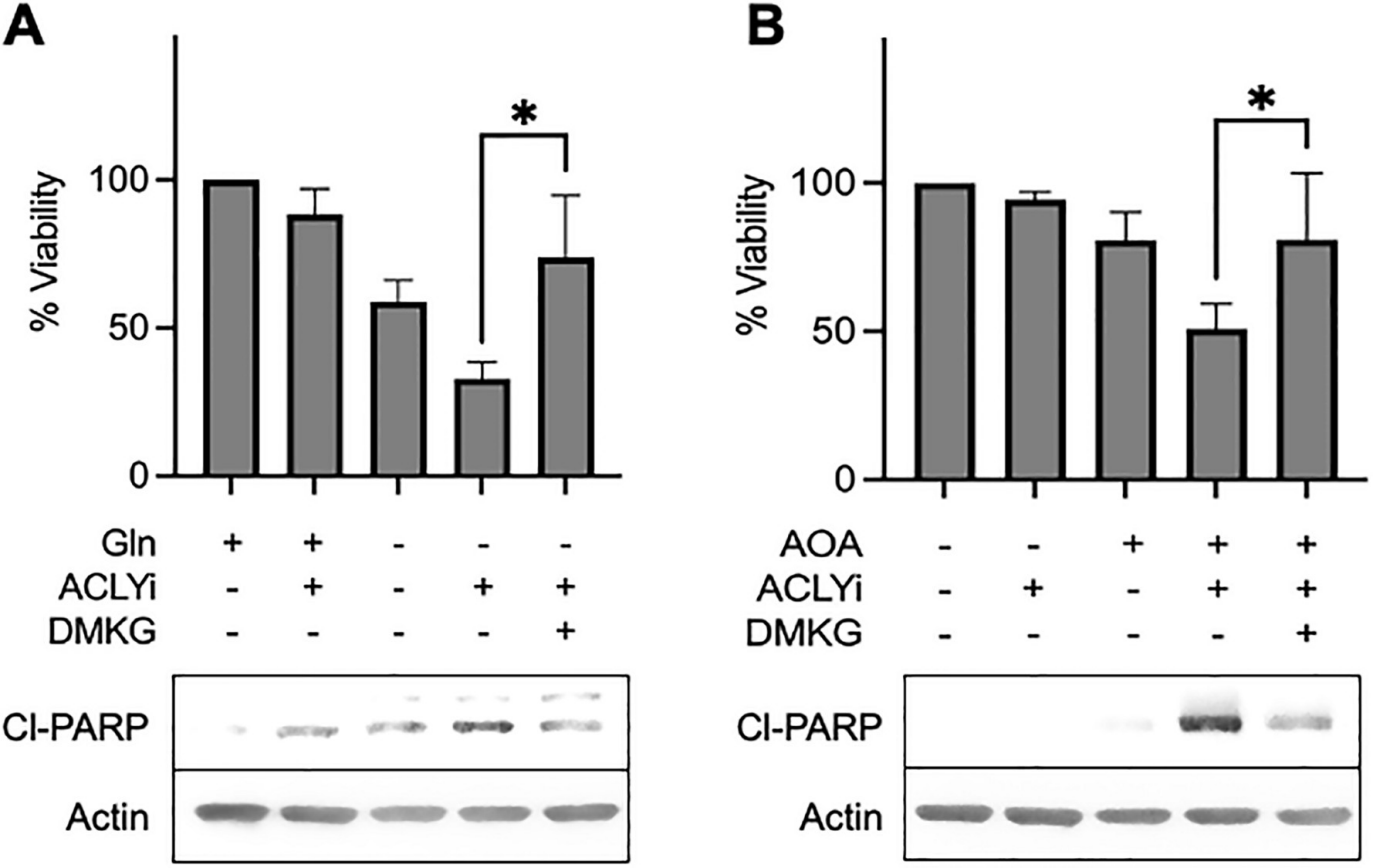
DMKG rescues cytotoxic effect of ACLY inhibition upon Gln deprivation or Gln utilization in KRas-driven cancer cells. (**A**) MDA-MB-231 cells were plated at 50% confluence in 96-well plates in complete medium (CM) containing 10% FBS. After 24 hr, cells were shifted to medium containing or lacking glutamine for 48 h. For the last 24 hr, cells were transferred into media containing SB-204990 (40 μM) with or without DMKG. Plates were read after 24 hr in plate reader and analyzed for cytotoxicity via XTT assay. Lysates were collected and analyzed for protein expression via western blotting. **(B**) MDA-MB-231 cells were plated as in *A*. After 24 hr, cells were shifted to medium containing or lacking AOA for 48 hr. For the last 24 hr, cells were transferred into media containing SB-204990 (40 μM) with or without DMKG (4 mM). Plates were read after 24 hr in plate reader and analyzed for cytotoxicity via XTT assay. Lysates were collected and analyzed for protein expression via western blotting. All experiments were repeated two or more times. Error bars represent ± SEM. Unpaired Student’s t-test was performed for statistical analysis. The Western blots shown are representative of experiments repeated at least two times.

The data in Fig 4B indicate that the inhibition of transamination reaction in glutaminolysis leads to loss of cell viability in KRas-driven cancer cells treated with the ACLY inhibitor SB-204990. The main products of the transamination reaction in glutaminolysis are α-KG and an amino acid such as aspartate from OAA or alanine from pyruvate [16, 19]. We therefore examined whether we could rescue the loss of cell viability caused by Gln deprivation or utilization and ACLY inhibition in KRas-driven cancer cells with α-KG. To do this, we added a cell-permeable analogue of α-KG–dimethyl-α-KG (DMKG). As shown in Fig 5A, depriving MDA-MB-231 cells of Gln in the presence the SB-204990 reduced cell viability and elevated PARP cleavage. If DMKG was included, the loss of cell viability was suppressed. The rescue of cell viability by DMKG was higher than the cell viability observed with Gln deprivation by itself–indicating that α-KG can rescue loss of viability induced by Gln deprivation.

We next investigated the ability of DMKG to rescue the loss of cell viability induced by the combination of the transaminase inhibitor AOA and the ACLY inhibitor SB-204990. As shown in Fig 5B, DMGK rescued the loss of cell viability induced by the combination of AOA and SB-204990. Under these conditions, the results were more striking in that AOA, by itself, did not reduce cell viability as was observed with Gln deprivation. These data suggest that the generation of α-KG via transaminase is important in KRas-driven cancer cells for cell viability.

## Discussion

**Fig 6:**
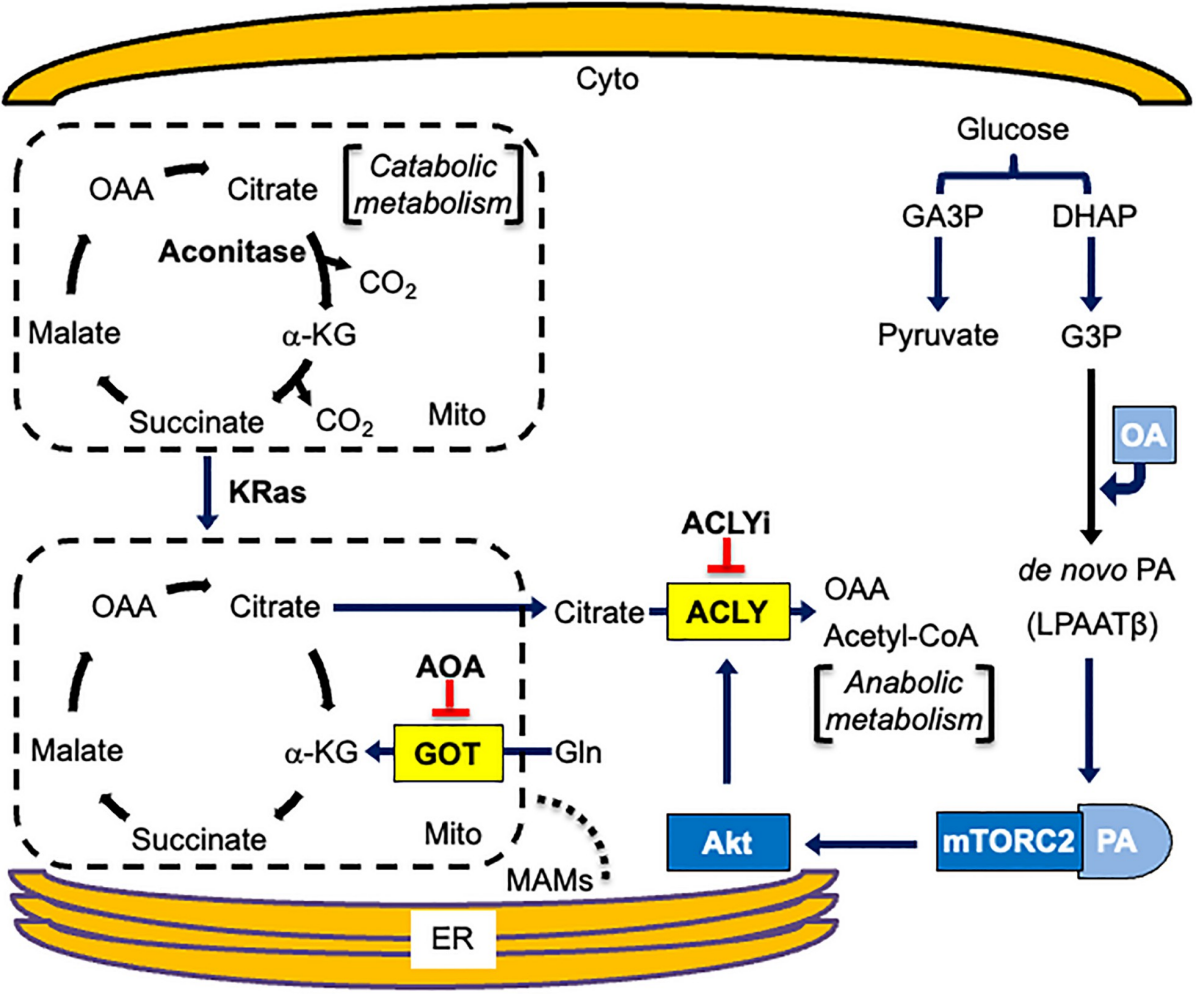
Anabolic and catabolic fates of citrate. Mitochondrial use of citrate is mainly catabolic via sequential oxidative decarboxylation reactions while cytosolic utilization of citrate yields anabolic precursors. Metabolic reprogramming in KRas-driven cancer cells changes the fate of citrate from mitochondrial catabolism to the anabolic ACLY pathway. Blocking ACLY in combination with blocking GOT disturbs the reprogramming in KRas-driven cancer cells creating a synthetic lethal situation where cancer cells harboring KRas mutations undergo apoptotic cell death. Additional abbreviations: ACLYi, ACLY inhibitor (SB-204990); Cyto, cytoplasm; DHAP, dihydroxyacetone phosphate; ER, endoplasmic reticulum; GA3P, glyceraldehyde 3-phosphate; G3P, glycerol 3-phosphate; Mito, mitochondrion.

In the work presented here, we provide evidence that the increase in mTORC2 (Akt phosphorylation at S473) in response to exogenously supplied OA and *de novo* synthesis of PA lead to the phosphorylation of ACLY in KRas-driven cancer cells. There is a metabolic reprogramming in KRas-driven cancer cells where glutaminolysis is used to generate the TCA cycle intermediate α-KG via a transaminase reaction [2, 6]. ACLY is activated by the mTORC2 substrate Akt–a kinase that phosphorylates ACLY at S455 [15]. Exogenously provided OA stimulates the phosphorylation of ACLY in a manner that is dependent on LPAAT-β, which generates PA, and mTORC2, which is activated by PA [12]. Activated ACLY catalyzes the breakdown of cytosolic citrate that has left the mitochondria to regenerate acetyl-CoA and OAA. In this way, acetyl-CoA generated from glycolysis can be used for the synthesis of fatty acids and other anabolic reactions [1]. Alternatively, mitochondrial citrate can be used for catabolic reactions whereby citrate is isomerized to isocitrate by aconitase and can be subjected to two oxidative decarboxylation reactions in the TCA cycle to generate two molecules of CO_2_ and two molecules of NADH that can be used in the electron transport chain to generate ATP. Thus, citrate generated by citrate synthase has two alternative fates–exiting the mitochondria where phosphorylated active ACLY regenerates acetyl-CoA and OAA, or remain in the mitochondria where aconitase isomerizes citrate to isocitrate, which can be decarboxylated twice to generate succinyl-CoA. The mitochondrial aconitase pathway is largely catabolic and leads to NADH production and ATP synthesis in the electron transport chain. The cytosolic ACLY pathway is largely anabolic and conserves the reduced carbons of acetyl-CoA. Similarly, Finley and colleagues recently described a “non-canonical” TCA cycle where the reduced acetyl-CoA was conserved by shuttling mitochondrial citrate to the cytosol where ACLY regenerates acetyl-CoA [31]. Importantly, ACLY is regulated by phosphorylation and, in principle, should drive the exit of citrate from the mitochondria by eliminating the cytosolic citrate by active phosphorylated ACLY that converts citrate to acetyl-CoA and OAA. Control of the fate of citrate–be it cytosolic and anabolic or mitochondrial and catabolic has profound consequences for cellular metabolism and the metabolic reprogramming that occurs in cancer cells. The two fates of citrate in the mitochondria and cytosol and regulation by ACLY is shown schematically in Fig 6.

The importance of ACLY in KRas-driven cancer cells is underscored by the sensitivity of these cells to the ACLY inhibitor SB-204990. We report here that SB-204990 treatment kills KRas-driven cancer cells in the absence of serum or deprivation of Gln. While the removal of either serum or Gln in the presence SB-204990 is synthetic lethal, this does not provide any viable therapeutic options. However, we were able to achieve a synthetic lethal phenotype using SB-204990 in combination with the pan transaminase inhibitor AOA that takes advantage of the observation that KRas-driven cancer cells are addicted to Gln [4] and use a transaminase pathway to generate α-KG from Gln-derived glutamate [2, 8, 12]. Importantly, AOA has been used in studies with mice, with no apparent toxicities [16, 17]. Thus, exploiting the metabolic reprogramming that takes place in KRas-driven cancers may permit the targeting of KRas, which has been difficult to target directly.

## Supporting information

S1 Raw images (PDF)
