# Supplementary material for: Inhibiting glutamine utilization creates a synthetic lethality for suppression of ATP citrate lyase in KRas-driven cancer cells
Source: PLoS One. 2022 Oct 21;17(10):e0276579. doi: 10.1371/journal.pone.0276579 (PMC9586366; doi:10.1371/journal.pone.0276579)
Supplement: S1 Raw images — (PDF) [file pone.0276579.s001.pdf]

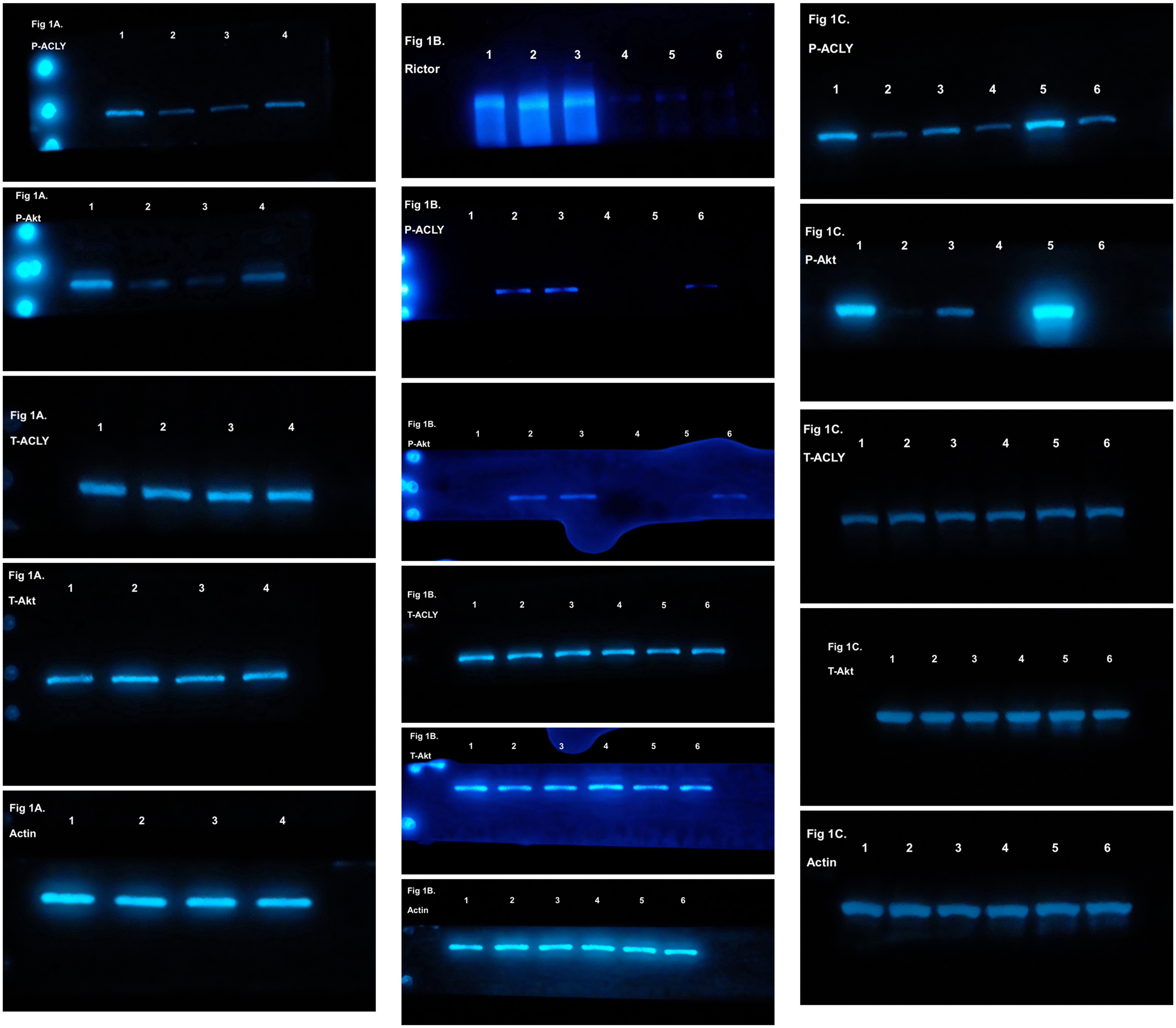

Fig 1. Oleic Acid induces ACLY phosphorylation in an mTORC2 dependent manner. See figure legend in manuscript for detailed description.

*For Fig 1C. Disregard the 5th and the 6th lanes. 5th lane refers to insulin and the 6th lane is insulin + Torin. We chose not to include these control samples in figure 1C.

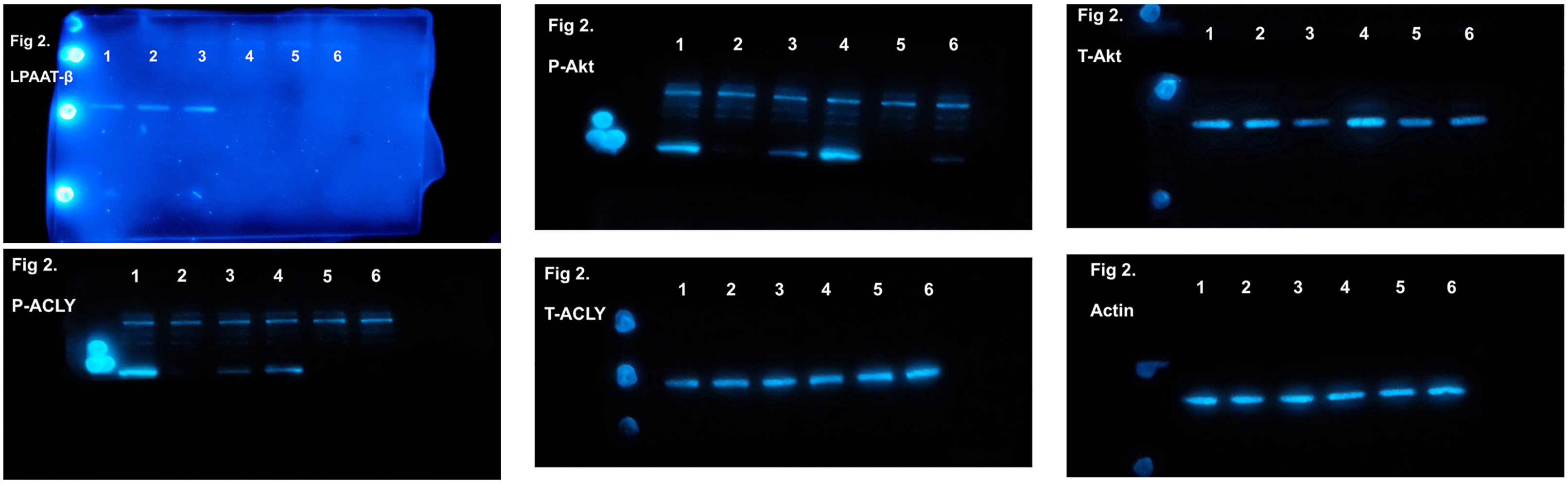

Fig 2. mTORC2 mediated activation of ACLY through production of de novo PA in presence of oleic acid. See figure legend in manuscript for detailed description.

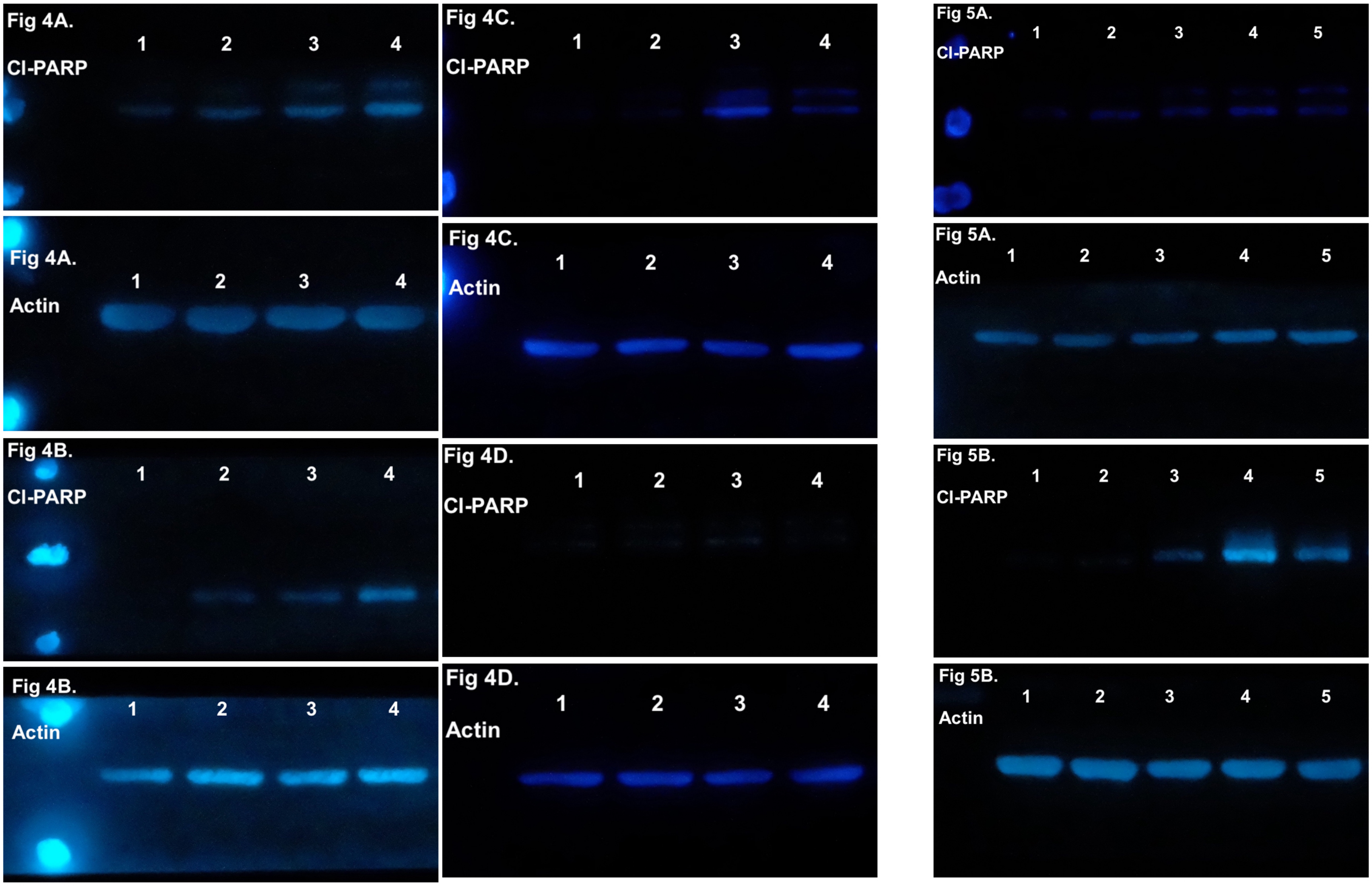

Fig 4. Glutamine deprivation sensitizes KRas-driven cancer cells to ACLY inhibition. See figure legend in manuscript for detailed description.

Fig 5. DMKG rescues cytotoxic effect of ACLY inhibition upon Gln deprivation or Gln utilization in KRas-driven cancer cells. See figure legend in manuscript for detailed description.
